# Performance study on a solar concentrator system for water distillation using different water nanofluids

**DOI:** 10.1016/j.heliyon.2023.e16535

**Published:** 2023-05-23

**Authors:** Hassanain Ghani Hameed, Hayder Azeez Neamah Diabil, Mohammed A. Al-fahham

**Affiliations:** a Engineering Technical College/Najaf, Al-Furat Al-Awsat Technical University, 31001, Najaf, Iraq; b Mechanical Dept., Engineering College, Kufa University, Najaf, Iraq

**Keywords:** Distilled water, Nanofluid, Solar energy, Solar concentrator system, Thermal efficiency

## Abstract

The rapid growth in the world–population urges the need for potable water in various regions, especially in hot and dry regions. The main challenge in the productivity of potable water is the cost and availability of water sources. Thus, it is crucial to develop effective methods to overcome this global need. Utilizing solar power is proven to be a promising path to implementing thermal solar radiation in solar distillation applications. This work investigates the effectiveness of using concentrated solar power to irradiate heat exchange to evaporate water in a receiver, which will be collected as pure water in a condenser later. The thermal performance of the proposed model and its productivity are tested experimentally by using tap water only, and the test was repeated twice using two nanofluids namely, (aluminium oxide (Al2O3) and zinc oxide (ZnO)). The results showed that using (Al2O3) has a superior influence on the productivity of the solar unit, where the productivity is increased by 43.53% and 21.89% when compared to tap water and zinc oxide (ZnO) nanofluid respectively. The thermal efficiency of the solar unit was also increased by 9.91% (maximum) when using (aluminium oxide (Al2O3) as a working fluid compared to tap water. The model has simple components and is easy to install with a compact size, which can be developed be utilized in urban and desert areas.

## 1 Introduction

Providing clean drinking water has crucial importance for humanity. It is well known that land wide areas are suffering from a limitation of pure water. However, these areas may involve groundwater, which is not suitable for drinking due to its high salinity. Water distillation is a costly process, especially when taking the environmental cost of fuel used. Moreover, the depletion of these resources motivates populations to attempt other methods to provide drinking water. One of these methods is using solar energy to provide thermal energy to distill brackish water because it is free of charge and pollution. Solar distillation is a proven method that uses radiant energy from the sun to purify saline water. With considerable low cost, absorbed solar energy evaporates impure water; consequently, water vapor separates from the dissolved matter and condenses in the form of pure water.

Practical applications that use solar energy may vary from heating water or air, obtaining high temperatures to generate steam for industrial purposes such as electricity generation, greenhouses, and cooking, to many other direct and indirect uses of solar energy depending on the method of harvesting it.

Many researchers [[1], [2], [3], [4], [5], [6]] are taking advantage of the availability of solar energy to purify water by distillation. Where the solar application differs from one researcher to another, depending on several factors, the most important of which is the cost and the efficiency of the application used. As a result of the low efficiency of the systems used, the researchers tended to use internal and external additives to improve efficiency and increase the yield from the solar system.

Ref. [7] reported many methods to use solar energy usefully; among these methods of converting solar energy is thermal transfer. The working principle of this method is to focus the solar radiation by concentrators on a thermal receiver to obtain high temperatures. Solar rays can be focused using mirrors and lenses. Mirrors are preferred because they are less expensive and lighter and absorb less energy than lenses due to focusing the rays by reflection. Generally, three types of solar thermal transfer systems have been employed in manufacturing fields: flat mirror concentrators, cylindrical mirror concentrators, and parabolic concentrators.

The parabolic solar concentrator is a mirror that is one or several pieces in the form of a parabola, as it focuses sunlight on the target called the receiver (heat absorber), which is placed in the focus. The target converts the solar radiation reflected from the mirrors into thermal energy to the fluid that passes inside the target, heating it to high temperatures, which allows it to be used in different applications.

The performance and productivity of solar concentrators have been investigated, analyzed, and developed in increasing studies to address the key parameters.

The following literature review sheds light on the efforts that have been carried out to develop different solar concentrator systems on the basis of their geometry, configurations, physics, using PCM and nano materials and many other parameters as following.

### 1.1 Solar concentrator system configuration

Ref. [8] theoretically and experimentally studied the performance of a laboratory solar desalination unit equipped with a parabolic concentrator. A theoretical model was developed to calculate the absorber temperature. The theoretical results show good agreement in terms of the average absorber temperature, but there was less agreement for the distillate flow rate. Ref. [9] built a low-cost dish solar concentrator made from an abandoned material in scrapyard. They reported good performance compared to the law value of construction. Ref. [10] extensively examined different materials of the solar reflector. Based on the solar reflector performance and durability, it was reported that glass, silvered polymer, and front-surface mirrors can remain for a 10-year lifetime. Performance and applications are detailed in [11] who conducted an experimental study on a Scheffler reflector with an area of 8 m^2^. The study shows that the overall efficiency of the absorber located in the center of the parabolic dish concentrator was 21.61%. Ref. [12] experimentally tested a cylindrical cavity receiver in a parabolic dish system. They argued that 52% optical efficiency and 4.6 W/K heat loss factor can be obtained using a cylindrical cavity receiver. Ref. [13] proposed a new design of a parabolic dish solar concentrator system. This design was characterized with a dual reflector. The additional reflector concentrated the solar energy to the engine generator located at the bottom of the primary reflector. The obtained heat flux and optical efficiency were 0.73 MW/m^2^ and 84.27%, respectively.

In terms of configuration, Ref. [14] experimentally and numerically studied a solar collector constructed from a parabolic dish reflector and spiral absorber. Three working fluids were employed in this study: water, therminol VP-1 and air. Results prove that water was the most efficient working fluid at low temperature levels, while at higher temperature values, the oil was the best. When the spiral receiver was replaced with a conical cavity shape absorber with a helical tube, the thermal performance of the system was enhanced with an average flux value of approximately 2.6 × 10^5^ W/m^2^. Ref. [15] experimentally investigated the change in the concentration ratio of a Scheffler concentrator. It was shown that the solar unit with a concentration ratio of 48 provided a 2-kW heat gain rate and steam with pressure up to 3 bar gauge pressure, while these parameters were 0.6 kW and up to 1 bar gauge pressure by using a Scheffler dish with a concentration ratio of 17. Ref. [16] numerically studied the effect of the configuration of the cavity receiver aperture on the natural convection heat loss in a solar parabolic dish concentrator system. Ref. [17] investigated the optimum operating conditions of a parabolic solar dish collector using a spiral coil absorber. The best results were 212.3° and 49.83% for the fluid temperature and thermal efficiency, respectively. Ref. [18] compared the operation of spiral and conical cavity absorbers in a solar parabolic dish concentrator. Due to the increase in the intercept factor, the results showed that the heat loss factor was 4.6 W/K and optical efficiency reached 52% with the use of the conical cavity receiver.

Ref. [19] Numerical analysis was implemented in this study using the Monte Carlo ray tracing method associated with the finite volume method. The authors reported that nonuniform heat flux from the tube surface was a result of a nonuniform distribution of temperature at the inner surface, and it was possible to largely reduce the surface heat losses using a quartz glass cover. Ref. [20] suggested a design to improve the performance of a tubular solar still that was located on the focus of a parabolic solar concentrator. The experimental results showed an increase in the tubular solar still productivity by 292.4%, and the efficiency was also enhanced by 82.3%. Ref. [21] argued that it is necessary to improve the geometry and surface properties of the solar receiver to improve the system performance. Therefore, they designed copper fins (conical shaped, tip/cone angle 30°) and fixed them inside a cavity surface with three different pitch values: 12.5 mm, 22 mm and 44 mm. The results of the new system showed that the solar performance was improved by 23.56% and 31.35% compared with the conventional system at medium and high temperature levels, respectively. For direct steam generation, Ref. [22] carried out an experiment in to evaluate the difference in the performance of a Scheffler concentrator with glazed and unglazed receivers. A tempered glass cover was employed on the aperture to reduce the radiation and convection losses from the receiver. The results showed that there was a reduction in the overall heat loss coefficient from 41.8 W/m^2^ K to 6.04 W/m^2^ K due to the use of the glazed receiver. In addition, 8.74% was recorded as an increase in the overall efficiency of the system provided by the receiver with a glass cover.

A comparison study was experimentally conducted in [23] to identify the best absorber among four types of absorbers in a solar parabolic dish concentrator system. The selected absorbers were a flat plate, disk, water calorimeter and solar heat exchanger. The results showed that the thermal energy efficiency ranged from 40% to 77%, while the recorded average exergy efficiency was 50%.

The performance of a parabolic dish solar concentrator unit was compared according to the change in the cavity receiver and working fluid [24]. Three receivers were chosen: hemispherical, cylindrical and cubical, while the employed working fluids were water and Behran oil. The authors presented that the best performance of the solar dish collector within the high temperature levels was achieved using the hemispherical cavity and oil, while water and the same receivers were the optimal parameters for higher thermal efficiency with lower temperatures values.

In the experiment of [25], the surface temperature of a 16 m^2^ Scheffler parabolic dish was recorded to be approximately 138–235 °C with an overall efficiency of 57.41%. Ref. [26] suggested a model to predict the temperature distribution on a solar volumetric receiver in a solar furnace. Two types of receivers were selected: molten salt and water/steam. Ref. [27] conducted an experiment to consume solar energy for cooking purposes. This was carried out using a parabolic dish solar concentrator with a cylindrical absorber and two working fluids as the heat transfer medium: water and synthetic oil. The average ambient temperature for the experimental period was 24 °C. The results showed that synthetic oil was more efficient than water, where the measured maximum temperature was 153 °C with synthetic oil against just 97 °C with water. Additionally, the maximum achieved energy was 29.0%.

Ref. [28] numerically proposed a new arrangement of a parabolic solar dish concentrator by dividing its surface into several parts and rotating each part around its one end. The ray tracing method and genetic algorithm were coupled within an integrated approach to improve the flux uniformity of the absorber surface inside a cavity receiver and optimize the solar dish concentrator. The results predicted a decrease in the nonuniformity factor by approximately 0.45, and the optical efficiency was between 88.93% and 92.19%. The work objective of [29] was to save electrical energy for a 1-ton room air conditioner by developing a solar refrigeration system. The designed solar-powered ejector refrigeration system was integrated with a flat-plate collector and Scheffler concentrator [30]. New design basics were using a Scheffler concentrator as a vapor system and an ejector as a cooling system. The authors argued that there was a possibility to reduce the consumed energy in the developed design by 70–80% compared with that in conventional air conditioners. Using the reflected ray from a parabolic solar concentrator to generate power and cooling from a solar-driven hybrid system. The results showed that the maximum average optical efficiency was 67.5%.

Ref. [31] uses thermoelectrical cooling system with solar dish concentrator to improve the fresh water production. The results showed that an increased up to 126% with a 36 W thermoelectric cooling capacity in potable water.

### 1.2 Using PCM and nano-fluid in solar concentrator systems

Ref. [32] conducted an experimental study to enhance the thermal efficiency of dish reflector using PCM, the authors reported 67.9% improvement in thermal efficiency of the system.

Ref. [33] conducted a numerical study to investigate the thermal efficiency of parabolic solar collector using two different water nanofluid. The properties of the water nanofluid are taken after prepared them in laboratory. Their outcome that using CuO improves the thermal efficiency by 14.79% at 0.0224 kg/s flow rate and the thermal efficiency was 13.1% when using Al_2_O_3_ at same flow rate. Ref. [6] show that Al_2_O_3_ has a better thermal efficiency compared to CuO and distilled water when are used to investigate the thermal efficiency of a conical concentrator system and have 72.5%.

Ref. [34] conducted an extensive review study to product clean water using small solar dish concentrator and solar still distillation systems. In this study the authors mentioned number of studies that use PCM (phase change material) to increase the water production and their conclusion of benefits of PCM.

In the literature, extensive efforts have been made to enhance the performance of solar concentrator systems. The distribution of temperature on the reflector focus and heat flux on the absorber surfaces have been concluded to play an important role in obtaining the maximum heat transfer and increasing the thermal performance of solar concentrator units. Many parameters have been investigated to increase the conversion efficiency of reflected solar energy to useful heat in working fluids, such as reflector type, reflector depth and how heat is distributed on the receiver.

To the best of the authors’ knowledge, using nanofluids as working fluid in a solar concentrator system and explore the effect of adding different nanoparticles on the system performance is very rear. The aim of the present work is to conduct experiments using couple of nanofluids that are known for their good thermal performance (aluminum oxide (Al_2_O_3_), zinc oxide (ZnO)) and compare the distilled water productivity from a parabolic dish solar concentrator system with and without nanofluids and shed light on how nanofluids improve the thermal performance of the system.

## 2 Solar unit fabrication

**Fig. 1:**
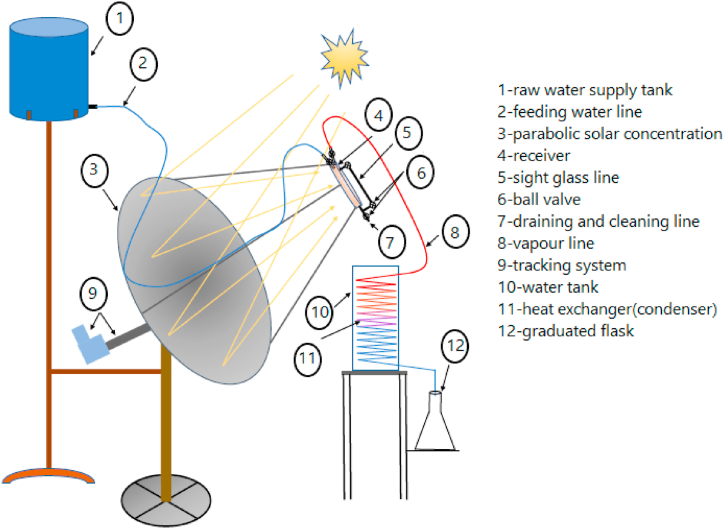
Sketch of the parabolic dish solar concentrator system km.

The construct of the solar unit in this work, benefits from previous studies in literature, and consists of a parabolic solar concentrator, receiver (heat absorber), water supply tank, condenser and sun tracking system as shown in Fig. 1. These components are detailed as follows:

The opening diameter and depth of the parabolic dish are 1.5 m and 0.17 m, respectively. Its inner surface is treated by covering it with 0.5 mm thick aluminum foil (reflecting coefficient near 0.8) as a reflecting layer. The reflected solar rays from the dish surface are concentrated on the face of the receiver that is located in the focal of the solar concentrator.

The receiver (heat absorber) is a stainless steel disc with dimensions of 0.25 m as diameter and 0.1 m as depth. All sides of the receiver except its face are covered with insulation to reduce the heat dissipation to the surroundings (2 cm thickness of white cork layer). To reduce the reflexional of the concentrated solar rays, the receiver face is painted with thermal black dye (absorption coefficient near 0.9). The water level inside the receiver is maintained according to the evaporation rate. The flow of the inlet water and outlet steam through the heat absorber is controlled using two ball valves fixed on the top of the receiver. A stainless steel tube with 3 mm diameter and 5 cm length was welded at the receiver base and turned toward the top. This pipe is connected with Pyrex pipe with 3 mm diameter and 20 cm length and used as a sight glass to point out the water level inside the receiver. Also, it is used to drain the concentrated salts due to the raw water distillation process at the end of the experiment day.

The raw (brackish) water is stored in a 25 L water tank, which placed in a level (2.5 m from the ground) higher than the maximum height that the receiver reaches during the experiment (1.98 m from the ground). The pressure head and water flow from the water tank to the receiver are controlled by a ball valve welded to the top of the receiver to ensure the desired water level inside the receiver. The water tank is connected to the heat absorber by a flexible tube with a diameter of 0.025 m joints with galvanized iron pipe, which is fixed on the holder that connects the center of the reflector and the receiver.

The delivered vapor from the receiver flows toward the condenser through a 0.01 m diameter aluminum pipe. Close to the condenser, part of this pipe is replaced with a flexible tube to allow the receiver to move up and down. The end of the aluminum pipe is constructed as a coil immersed inside a plastic container that is filled with water under ambient temperature to produce pure water. Eventually, the distilled water yield is accumulated in a scaled flask located outside the condenser to measure the water productivity.

A sun tracking system to rotate the dish is locally constructed. Its performance depends on an electric motor with remote control that is used to track the satellite systems. One end of the motor is connected to the backside of the dish and the other end is connected to the mechanism stand. The motor is connected to an electronic tracking unit that is constructed locally to rotate the dish one degree in the east–west direction every 5 min. This ensures a continuous reflection of the solar rays to the focal zone. To adjust the correct focal point, the dish is manually rotated toward the sun declination in the north–south direction on the day of the experiment.

## 3 Mathematical background

**Fig. 2:**
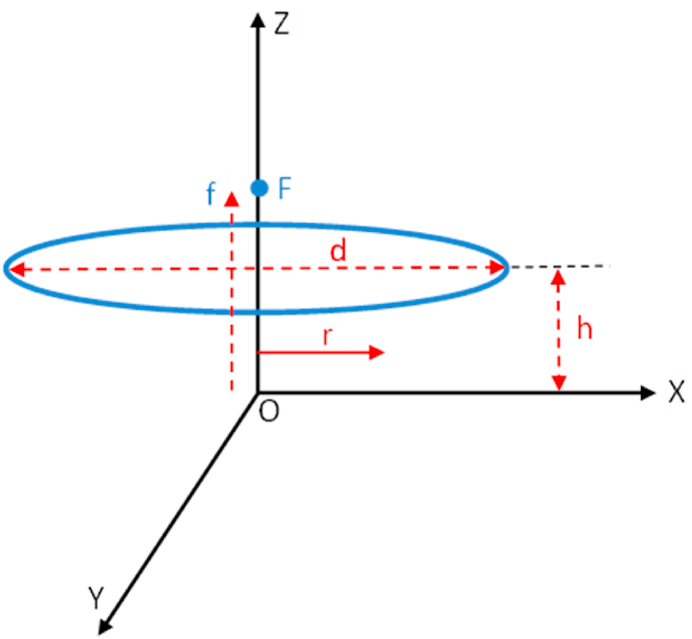
Geometric parameters of a parabolic dish.

The basic mathematical background of the present study was discussed in [35]. A schematic diagram of a parabolic dish is shown in Fig. 2.

The equation for the parabola in the cylindrical coordinates is [35]:

(1) $$z=\frac{r^{2}}{4f}$$

where f is the focal length of the parabola. According to the focal length and diameter of the opening parabolic surface d, the surface of this parabola is given by:

(2) $$S=\frac{8\pi }{3}f^{2}\left[{\left(1+{\left(\frac{d}{4f}\right)}^{2}\right)}^{3/2}-1\right]$$

The cross-section of the opening parabola is:

(3) $$S_{0}=\frac{\pi d^{2}}{4}$$

The focal length is identified from:

(4) $$f=\frac{d^{2}}{16h}$$

where h is the depth of the dish.

The geometrical concentration of the current receiver is:

(5) $$C_{g}=\frac{S_{0}}{S_{a}}$$

where $S_{a}$ is the lighted area of the heat absorber.

The characteristics of the parabolic dish solar concentrator and heat absorber used in the experiments are detailed in Table 1, Table 2, respectively.

**Table 1:** Characteristics of the parabolic dish solar concentrator.

Diameter of opening of the parabola d	1.5 m
Depth of the parabola h	0.17 m
Total surface of the parabola S	1.86 m^2^
Surface collecting of the parabola $S_{0}$	1.76 m^2^
Focal length f	0.827 m

**Table 2:** Characteristics of the heat absorber.

Material	Stainless steel
Receiving diameter	0.25 m
Depth	0.1 m
Receiving surface	0.049 m^2^
Geometrical concentration factor $C_{g}$	35.918
Thermal conductivity	20 W/m^.^K

## 4 Working nanofluids

To improve the thermal conductivity of fluids, nanoparticles are used as a solution. To select the optimal nanopowder to produce the nanofluids, two factors must be considered: thermal conductivity and cost of nanoparticles. Based on [36,37] aluminum oxide (Al_2_O_3_), zinc oxide (ZnO), iron oxide (Fe_2_O_3_) and tin oxide (SnO_2_) are less expensive. Therefore, these nanoparticles are the best choice for nanofluid production even though their thermal conductivity is less than that of other nanoparticles, such as carbon nanotubes, diamond nanopowder (C), gold nanopowder (Au) and others that have high cost.

**Fig. 3:**
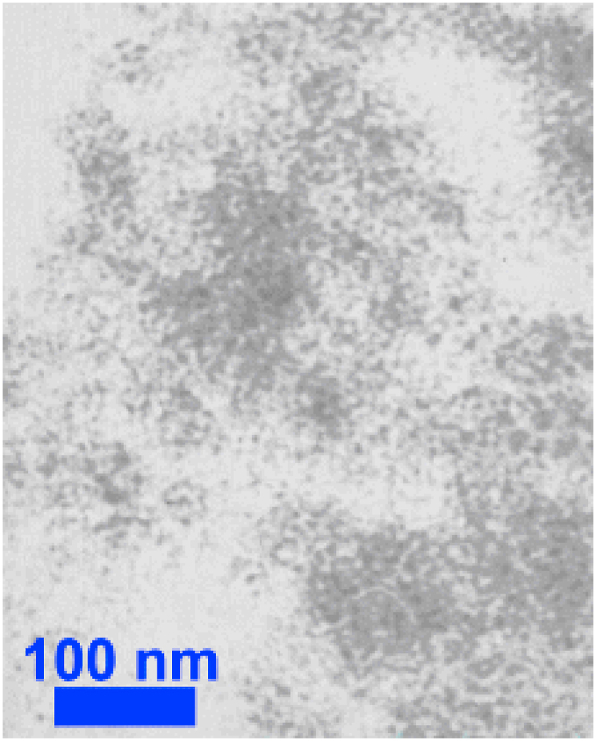
TEM micrograph of nano-alumina.

**Fig. 4:**
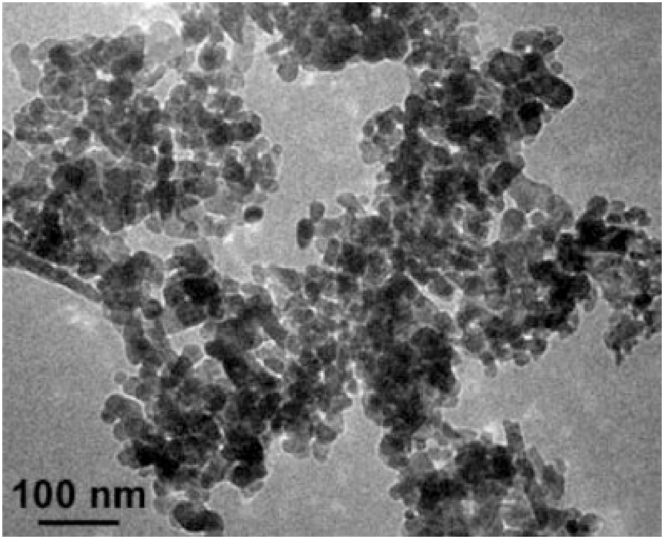
TEM micrograph of ZnO.

In the present study, two types of nanoparticles, Al_2_O_3_ and ZnO, are used to produce nanofluids. The specifications of the Al_2_O_3_ and ZnO nanoparticles and TEM are shown in Table 3 and Fig. 3, Fig. 4.

**Table 3:** Properties of the nanoparticles.

	Al_2_O_3_	ZnO
Purity %	99.97	99.5
Particle Size	20 ± 5 nm	25 ± 5 nm
Density	3890 kg/m^3^	5600 kg/m^3^
Thermal Conductivity	40 W/m.K	29 W/m.K
Specific heat	880 J/kg. K	494 J/kg. K
Particle Morphology	nearly spherical	nearly spherical
Source	US Research Nanomaterials, Inc. USA	US Research Nanomaterials, Inc. USA

The current nanofluids were locally produced in the labs of Engineering Collage, Kufa University, Iraq. A two-step method is conducted to produce the nanofluids. The nanofluid was produced by direct dispersion of the nanoparticles of Al_2_O_3_ or ZnO into the water in a Pyrex flask that can be sealed by a PVC cap. The flask was placed in a stainless steel basket inside an ultrasonic water bath (type Elmasonic P180H and supplied by Elma, Germany). The ultrasonic device basin was filled with water over the mixture level in the flask by approximately 3 cm. To remove air from the mixture, the next step that represented by switching on the degas mode was carried out. Then, the cap was used to seal the flask, which continuously oscillated for 10 h for Al_2_O_3_ and 12 h for ZnO in an ultrasonic water bath with a frequency of 37 kHz and a power efficiency of 100% at 60–70 °C. Therefore, the nanoparticles can be uniformly dispersed. It is worth pointing out that there was no addition of surfactants added into the nanofluid to avoid their effects on the thermophysical properties of the nanofluid. The container of the ultrasonic bath was sealed with a cap to avoid the evaporation of water during sonication.

To calculate the properties of the nanofluids, the correlations presented in [38,39] are used as follows:

(6) $$\frac{1-w}{w}\times \frac{\rho_{n}}{\rho_{f}}=\frac{1-\varphi }{\varphi }$$

In Equation (6), the nanofluid volume fraction can be calculated based on the nanofluid mass concentration. In the present work, the nanofluid volume fraction for both Al_2_O_3_ and ZnO is 0.002.

The density of the nanofluid is evaluated from [38,39]:

(7) $$\rho =\left(1-\varphi \right)\rho_{f}+\varphi \rho_{n}$$

The specific heat formula of the nanofluid is given by:

(8) $$c_{p}=\left(1-\varphi \right)c_{pf}+\varphi c_{pn}$$

The thermal conductivity of the nanofluid is calculated from:

(9) $$k=k_{f}\frac{k_{n}+2k_{f}-2\left(k_{f}-k_{n}\right)\varphi }{k_{n}+2k_{f}+\left(k_{f}-k_{n}\right)\varphi }$$

The calculated thermal conductivity of the nanofluids and the weight of the nanoparticles used is shown in Table 4.

**Table 4:** Thermal conductivity of the current nanofluids.

Nanofluid	Thermal conductivity (W/m^.^K)	Weight of nanoparticles (g)
Al_2_O_3_	0.652	199
ZnO	0.631	280

## 5 Experimental methodology

Three experiments were carried out on three consecutive days starting from May 2, 2021. The first day was for the solar unit using water as a working fluid. The second and third days were for the solar unit using Al_2_O_3_ and ZnO water nanofluids, respectively. The location of all experiments was in Najaf city/Iraq (latitude and longitude are 32° 03′ N and 44° 19′ E).

Four K-type calibrated thermocouples with efficient insulation are distributed along the solar system and connected to a multichannel digital display unit (Applent digital datalogger AT-4532x) to measure, hourly, the temperatures of the inlet water, inside water and outlet vapor of the heat absorber and the ambient. Solar radiation was measured at each hour of the experiment by a TENMARS solar power meter (TM-207). The wind speed was taken hourly by an anemometer device type (AM-4206 M). The accuracy and range for the measuring instruments are detailed in Table 5. The standard uncertainties are determined by considering linear variation in the data of equipment, and thus, it is considered to be *a*/√3 where a is the accuracy of the measuring instrument [40].

**Table 5:** Uncertainty and accuracy for the measuring instruments.

Instruments	Accuracy	Range	Standard Uncertainty
Thermocouples	±1 °C	0–280 °C	0.577 °C
Solar power meter	±10 W/m^2^	0–2000 W/m^2^	5.77 W/m^2^
Anemometer	±0.1 m/s	0–30 m/s	0.057 m/s
Scaled flask	±10 ml	5000 ml	5.77 ml

The measured ambient conditions were recorded each hour within the experimental interval (between 8.00 and 18.00). It is worth pointing out that there was no considerable change in the magnitudes of the hourly ambient conditions, especially for the values of the ambient temperature and solar radiation, on the second and the third experimental days compared with that on the first experimental day.

Raw water in the water tank (with a salinity of 1850 ppm) is passed into the receiver through the ball valve to fill it until a known level is reached. The reflected rays from the solar concentrator surface on the face of the receiver will heat the water, converting it to vapor, which escapes from the receiver by the other ball valve toward the condenser. Eventually, the pure water is accumulated in a scaled flask and measured at each hour of the experiment. The desired water quantity within the heat absorber must be maintained during the experiment by regularly removing the salt deposit in the heat absorber.

## 6 Thermal efficiency

As presented in [12] the thermal efficiency of the solar collector system ($\eta_{th}$) is the ratio of the useful heat production ($Q_{u}$) to the solar radiation on the dish aperture ($Q_{s}$).

(10) $$\eta_{th}=\frac{Q_{u}}{Q_{s}}$$

The useful heat production ($Q_{u}$) can be obtained by balancing the energy in the fluid volume as:

(11) $$Q_{u}=\dot{m}c_{p}\left(T_{out}-T_{in}\right)$$

The value of solar energy ($Q_{s}$) is:

(12) $$Q_{s}=S_{o}I$$

In the present work, the hourly water mass flow rate is estimated according to the amount of pure water produced at each hour of the experiment.

## 7 Results and discussion

**Fig. 5:**
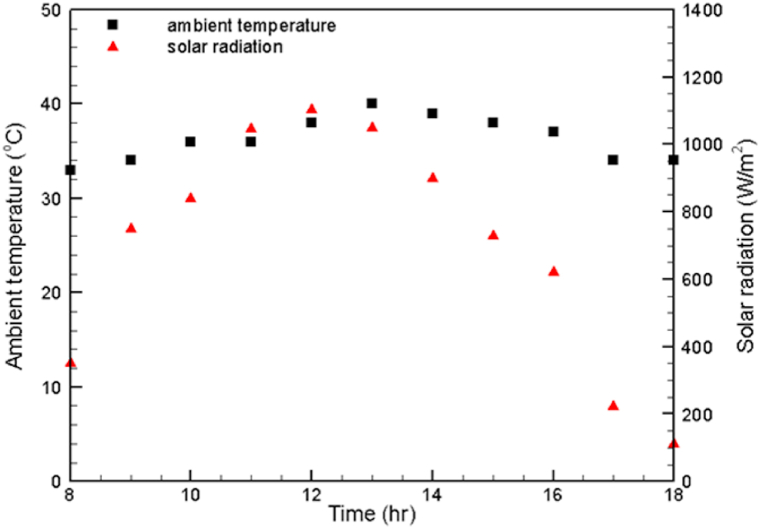
Variations in the ambient temperature and solar radiation on May 2, 2021.

**Fig. 6:**
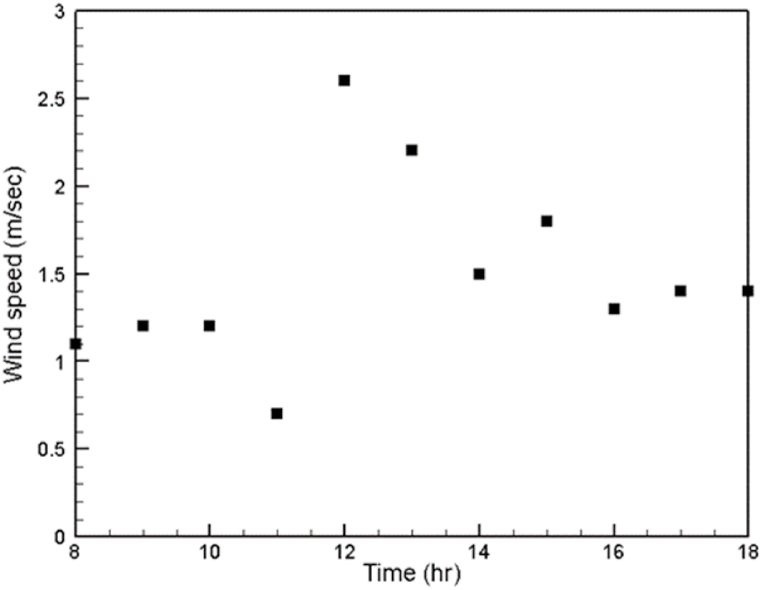
Variation in the wind speed on May 2, 2021.

The variation in the values of the measured climatic conditions during the experimental period is illustrated in Fig. 5, Fig. 6. It is worth pointing out that even though the experiments were carried out on different days (2, 3, 4/5/2021), there was an insignificant change in the magnitudes of the ambient conditions at each experimental hour on each experimental day, especially in values of the ambient temperature and solar radiation. Therefore, only the solar radiation, wind speed and atmospheric temperature of the first experimental day are presented here. In Fig. 5, it is clearly seen that the maximum value of the solar radiation is 1103 W/m^2^ at midday. Then, the solar radiation value dramatically decreases, reaching 112 W/m^2^ at 6 p.m. Fig. 5 also shows that during the experimental time, the difference between the magnitudes of the maximum and minimum ambient temperatures is 7 °C. Fig. 6 clearly shows that the highest change in the wind speed on May 2, 2021 is 1.9 m/s.

**Fig. 7:**
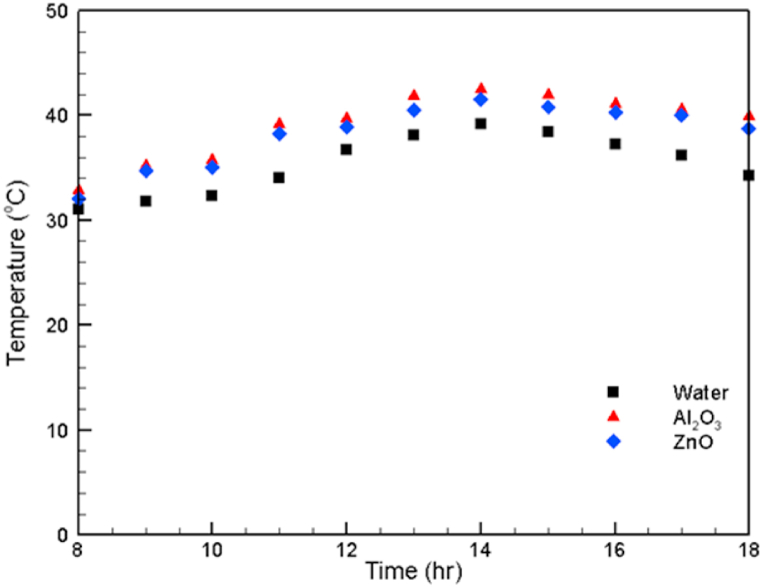
Variation in the tank fluid temperature.

The variation in fluid temperature that flows from the tank to the receiver is shown in Fig. 7. The thermal conductivity of the fluid affects its temperature. At each hour of the experiment, the highest temperature is for the aluminum oxide (Al_2_O_3_) nanofluid, followed by the zinc oxide (ZnO) nanofluid and then water. For all fluids, the highest temperature was recorded at 2 p.m.

**Fig. 8:**
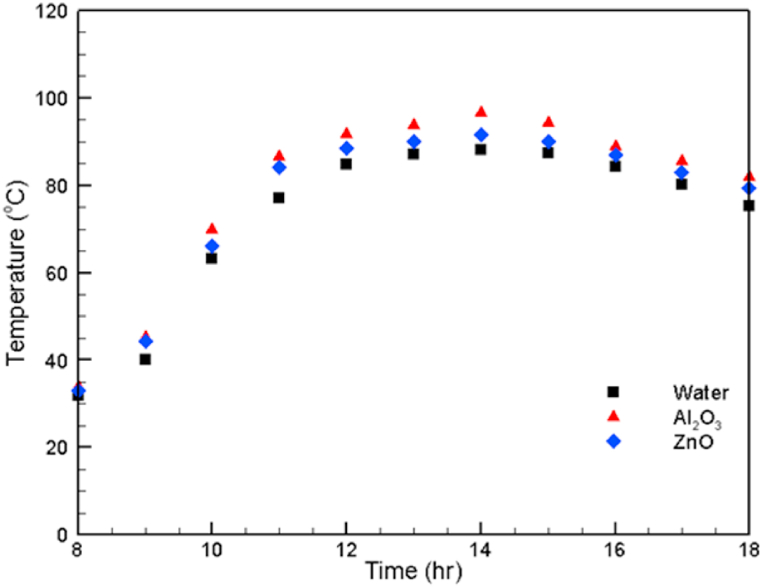
Variation in the value of the receiver fluid temperature.

Fig. 8 presents the change in the heat absorber fluid temperature. It is clearly shown that the highest temperature of the highest thermal conductive fluid (Al_2_O_3_ nanofluid) is 96.5 °C. This temperature is more than the highest temperature of the ZnO nanofluid by 5 °C. It is also seen that the highest temperature of water, which is the lowest thermal conductive fluid, does not exceed 88.2 °C, as shown in Fig. 8. It can be seen that the dependency on the fluid thermal conductivity also appears in the inspection of the temperature of the heat absorber fluid.

**Fig. 9:**
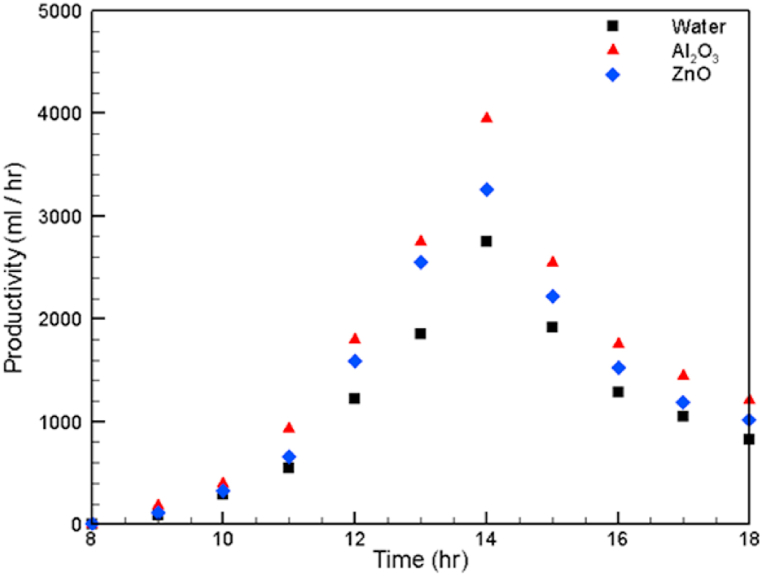
Variation in fresh water productivity.

The production rate of pure water is shown in Fig. 9. It can be observed that distilled water productivity is higher for fluids with higher thermal conductivity. This is a consequence of the higher thermal energy conducted due to the earlier heating of the Al_2_O_3_ nanofluid. It is clearly seen that the maximum amount of fresh water produced at 2 p.m. is 3950 ml, 3250 ml and 2750 ml for the Al_2_O_3_ nanofluid, ZnO nanofluid and water, respectively. Therefore, the rate of pure water productivity is also a function of the fluid thermal conductivity.

**Fig. 10:**
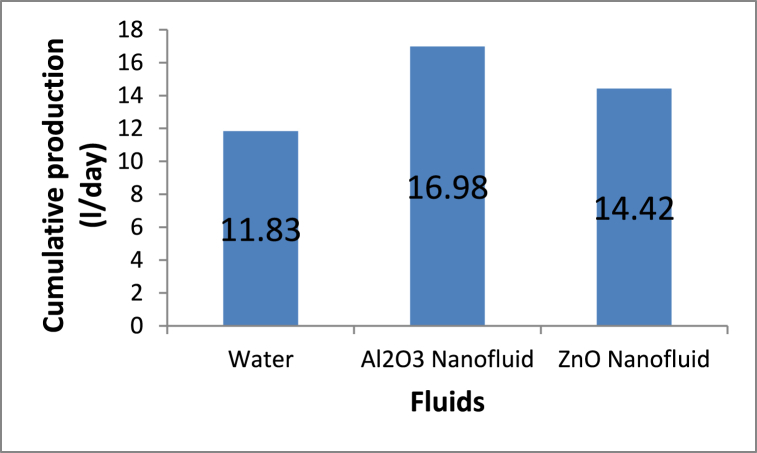
Cumulative production of fresh water.

An increase in the thermal conductivity leads to more cumulative production of fresh water, as shown in Fig. 10. Using an Al_2_O_3_ nanofluid as a working fluid in the solar concentrator system produces 16.98 l/day. This amount of water decreases with decreasing thermal conductivity of the employed fluids, as observed in Fig. 10.

**Fig. 11:**
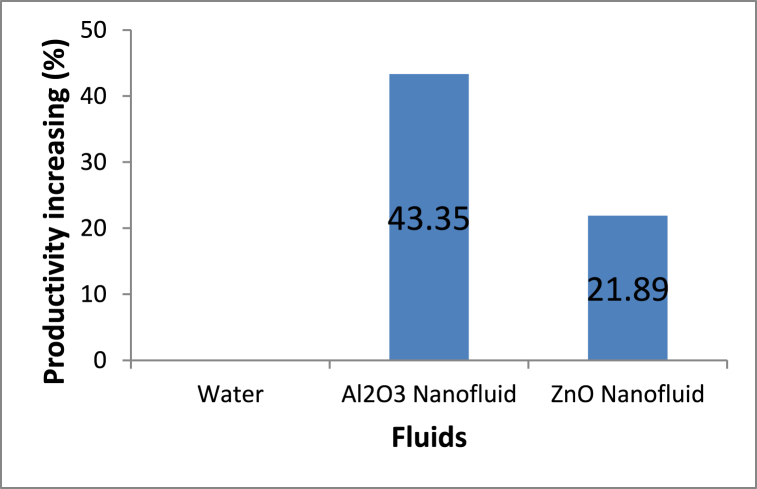
Increasing percentage in fresh water production of nanofluids compared with water.

The increasing percentage in the total production of the current solar concentrator system due to the use of various fluids is presented in Fig. 11. The fresh water produced per day increased due to the use of fluids with higher thermal conductivity. The total production of the solar concentrator unit with the Al_2_O_3_ nanofluid exceeds 43.35% compared with the conventional unit that uses ordinary water. This ratio decreases to 21.89% with the use of the ZnO nanofluid due to its lower thermal conductivity compared with that of the Al_2_O_3_ nanofluid.

**Fig. 12:**
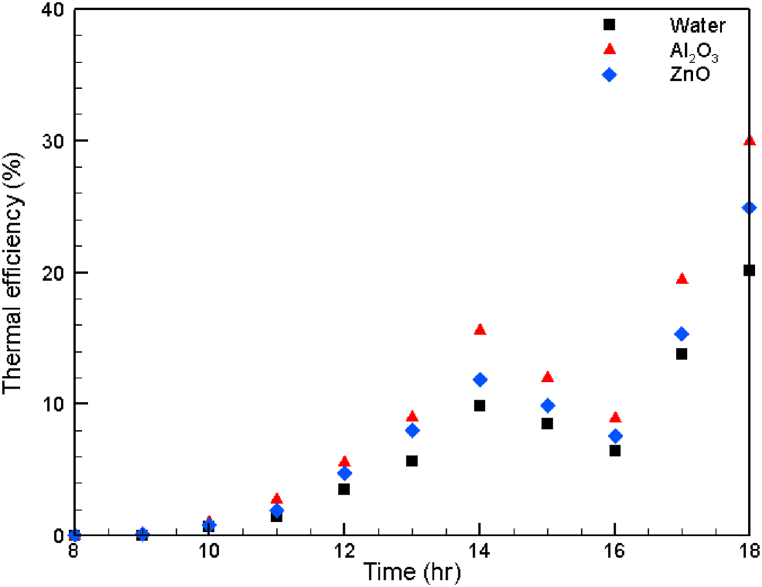
Variation in the thermal efficiency.

The calculated thermal efficiency of the solar unit is shown in Fig. 12. This figure shows the change in the thermal efficiency of the solar unit due using of various fluids during the experimental time. It is clearly shown that the thermal efficiency of the solar unit with any current fluid increases for intervals from 8 a.m. to 2 p.m. Until mid-day, no significant difference in the thermal efficiency can be noticed by a comparison among the results of the three current fluids. However, the thermal efficiency that comes from using the Al_2_O_3_ nanofluid is still the highest. Indeed, this is due to the higher thermal conductivity of this fluid. At the time interval between 12 and 2 p.m., the effect of thermal conductivity clearly appears. At 2 p.m., the thermal efficiency of the solar unit with the Al_2_O_3_ nanofluid is higher than that with ordinary water by approximately 5.74%. In the period of 2–4 pm, there is a reduction in the thermal efficiency of the solar unit for all fluids due to the reduction in the useful heat production that is a consequence of the decrease in the rate of freshwater production. After 4 p.m., the thermal efficiency of the solar unit with each current fluid dramatically increases, reaching its maximum value at the end of the experiment day. The dramatic increase in the thermal efficiency is due to the sharp reduction in the solar energy, which is a result of the decrease in the solar radiation between 4 and 6 p.m. In addition, through this time, the useful heat production is still high because of the high temperature difference of the working fluid. In general, the maximum thermal efficiency of the solar unit due to using the Al_2_O_3_ nanofluid is 30%, which is higher than that obtained from using the ZnO nanofluid and water by 5.11% and 9.91%, respectively.

In general, the variation in the experimental results with the local time is shown in Table 6.

**Table 6:** Variation in the experimental results with the local time.

Time (hr)	Tank fluid temperature (°C)			Receiver fluid temperature (°C)			Fresh water productivity (ml/hr)			Thermal efficiency %		
	Water	Water-Al_2_O_3_	Water-ZnO	Water	Water-Al_2_O_3_	Water-ZnO	Water	Water-Al_2_O_3_	Water-ZnO	Water	Water-Al_2_O_3_	Water-ZnO
8	31	32.9	32	32	33.9	32.8	0	0	0	0	0	0
9	31.8	35.2	34.7	40.1	45.3	44.3	85	180	110	0.062	0.159	0.092
10	32.3	35.8	35	63.3	69.8	66.1	290	400	320	0.706	1.065	0.780
11	34	39.2	38.2	77.1	86.5	84.1	550	930	650	1.496	2.782	1.890
12	36.7	39.7	38.9	84.7	91.7	88.4	1220	1800	1590	3.502	5.588	4.690
13	38.1	41.8	40.5	87.1	93.8	90	1850	2750	2550	5.695	8.976	7.938
14	39.2	42.5	41.5	88.2	96.5	91.5	2750	3950	3250	9.877	15.618	11.872
15	38.4	42	40.8	87.4	94.2	90	1920	2550	2220	8.502	11.996	9.870
16	37.2	41.1	40.3	84.2	88.8	86.9	1285	1760	1520	6.426	8.904	7.549
17	36.2	40.6	40	80.2	85.6	83.1	1050	1450	1190	13.791	19.478	15.380
18	34.3	39.9	38.8	75.4	82	79.5	830	1210	1020	20.093	30.006	24.897

## 8 Conclusions

The availability of fresh water is a very important factor to ensure the continuity of human life. Many efforts have been made to improve different methods to reduce the cost of fresh water productivity. One of these methods is using solar energy to evaporate brackish water, which is condensed later to produce pure water. The solar concentrator system is one of the solar energy applications. In the literature, the performance of this system has been improved using different enhancement methods. However, using nanofluids as additives to enhance the evaporation of brackish water has not been studied extensively.

In the current work, it is observed that the productivity of pure water and the thermal performance of the solar concentrator system increase due to the use of different water nanofluids. Two nanofluids are selected: aluminum oxide (Al_2_O_3_) and zinc oxide (ZnO). The thermal efficiency and freshwater production are functions of the fluid thermal conductivity. Due to high thermal conductivity of the Al_2_O_3_ and ZnO nanofluids are used as working fluids in the solar system increase operating temperature up to a maximum value of 96.5 °C for Al_2_O_3_ and less by 5 °C for ZnO at 2 p.m. Also, this increase the distilled water production by 5.15 and 2.59 l/day for Al_2_O_3_ and ZnO respectively, compared with the amount of fresh water that is produced using ordinary water in the conventional solar system. In addition, the difference in the thermal conductivity between the Al_2_O_3_ nanofluid and ZnO nanofluid leads to an increase in the freshwater production rate by 21.46%. The thermal efficiency of the solar concentrator system for each working fluid is calculated and investigated. It is shown that the maximum thermal efficiency of the solar unit due to using Al_2_O_3_ nanofluid is 30%, which exceeds by 5.

## Author contribution statement

Hasnain Hameed: Conceived and designed the experiments; Performed the experiments.

Mohammed Al-fahham: Analyzed and interpreted the data; Contributed reagents, materials, analysis tools or data; Wrote the paper.

Hayder Azeez Neamah Diabil: Conceived and designed the experiments; Performed the experiments; Analyzed and interpreted the data; Wrote the paper.

## Data availability statement

Data included in article/supplementary material/referenced in article. Any further need for data and material authors are ready to upload when be asked.

## Declaration of competing interest

The authors declare that they have no known competing financial interests or personal relationships that could have appeared to influence the work reported in this paper.

## Nomenclature

$c_{p}$: Specific heat capacity under constant pressure, J/kg K
$c_{pf}$: Base fluid specific heat, J/kg K
$c_{pn}$: Nanoparticles specific heat, J/kg K
I: Solar radiation, W/m^2^
k: Nanofluid thermal conductivity, W/m^.^K
$k_{f}$: Base fluid thermal conductivity, W/m^.^K
$k_{n}$: Nanoparticles thermal conductivity, W/m^.^K
$\dot{m}$: Fresh water mass flow rate, kg/s
$T_{in}$: Temperature of fluid which flows from the water tank, K
$T_{out}$: Temperature of fluid inside the receiver, K
*W*: Nanofluid mass concentration, -
φ: Nanofluid volume fraction, -
ρ: Nanofluid density, kg/m^3^
$\rho_{f}$: Base fluid density, kg/m^3^
$\rho_{n}$: Nanoparticles density, kg/m^3^

## References

[1] K. A. E, Sathyamurthy R. (2018). Different parameter and technique affecting the rate of evaporation on active solar still-a review. Heat Mass Tran..

[2] Kabeel A.E. (2019). Effect of water depth on a novel absorber plate of pyramid solar still coated with TiO2 nano black paint. J. Clean. Prod..

[3] Athikesavan M.M., Márquez F.P.G., Rafeek M.T.M., Sathyamurthy R. (2021). Proceedings of the Fifteenth International Conference on Management Science and Engineering Management.

[4] Benoudina B., Attia M.E.H., Driss Z., Afzal A., Manokar A.M., Sathyamurthy R. (2021). Enhancing the solar still output using micro/nano-particles of aluminum oxide at different concentrations: an experimental study, energy, exergy and economic analysis. Sustain. Mater. Technol..

[5] Rafiei A., Loni R., Mahadzir S.B., Najafi G., Pavlovic S., Bellos E. (2020). Solar desalination system with a focal point concentrator using different nanofluids. Appl. Therm. Eng..

[6] Abdalha Mahmood A.H., Hussain M.I., Lee G.-H. (2022). Effects of nanofluids in improving the efficiency of the conical concentrator system. Energies.

[7] Duffie J.A., Beckman W.A., McGowan J. (1985). Solar engineering of thermal processes. John Wiley & Sons.

[8] Chaouchi B., Zrelli A., Gabsi S. (2007). Desalination of brackish water by means of a parabolic solar concentrator. Desalination.

[9] Palavras I., Bakos G.C. (2006). Development of a low-cost dish solar concentrator and its application in zeolite desorption. Renew. Energy.

[10] Kennedy C.E., Terwilliger K. (2005). Optical durability of candidate solar reflectors. J. Sol. Energy Eng..

[11] Patil R., Awari G.K., Singh M.P. (2011). Experimental analysis of Scheffler reflector water heater. Therm. Sci..

[12] Mawire A., Taole S.H. (2014). Experimental energy and exergy performance of a solar receiver for a domestic parabolic dish concentrator for teaching purposes. Energy Sustain. Dev..

[13] Wardhana A.S., Suryoatmojo H., Ashari M. (2016). 2016 International Seminar on Intelligent Technology and its Applications (ISITIA).

[14] Pavlovic S., Daabo A.M., Bellos E., Stefanovic V., Mahmoud S., Al-Dadah R.K. (2017). Experimental and numerical investigation on the optical and thermal performance of solar parabolic dish and corrugated spiral cavity receiver. J. Clean. Prod..

[15] Nene A., Suyambazhahan S., Ramchandran S. (2017). Comparative analysis of performance of two Scheffler solar concentrators having different concentration ratios. Int. J. Eng. Technol..

[16] Tao Y.B., He Y.L., Cui F.Q., Lin C.H. (2013). Numerical study on coupling phase change heat transfer performance of solar dish collector. Sol. Energy.

[17] Stefanovic V.P., Pavlovic S.R., Bellos E., Tzivanidis C. (2018). A detailed parametric analysis of a solar dish collector. Sustain. Energy Technol. Assessments.

[18] Kumar N.S., Reddy K.S. (2008). Comparison of receivers for solar dish collector system. Energy Convers. Manag..

[19] Mao Q., Shuai Y., Yuan Y. (2014). Study on radiation flux of the receiver with a parabolic solar concentrator system. Energy Convers. Manag..

[20] Elashmawy M., Alshammari F. (2020). Atmospheric water harvesting from low humid regions using tubular solar still powered by a parabolic concentrator system. J. Clean. Prod..

[21] Bopche S., Rana K., Kumar V. (2020). Performance improvement of a modified cavity receiver for parabolic dish concentrator at medium and high heat concentration. Sol. Energy.

[22] Malwad D., Tungikar V. (2020). Thermal performance analysis of glazed and unglazed receiver of scheffler dish. J. Therm. Eng..

[23] Skouri S., Bouadila S., Ben Salah M., Ben Nasrallah S. (2013). Comparative study of different means of concentrated solar flux measurement of solar parabolic dish. Energy Convers. Manag..

[24] Abid M., Khan M.S., Ratlamwala T.A.H., Amber K.P. (2020). Thermo environmental investigation of solar parabolic dish assisted multi generation plant using different working fluids. Int. J. Energy Res..

[25] Dafle V.R., Shinde N.N. (2012). Design, development & performance evaluation of concentrating monoaxial Scheffler technology for water heating and low temperature industrial steam application. Int. J. Eng. Res. Afr..

[26] Roldán M.I., Monterreal R. (2014). Heat flux and temperature prediction on a volumetric receiver installed in a solar furnace. Appl. Energy.

[27] Mbodji N., Hajji A. (2016). Performance testing of a parabolic solar concentrator for solar cooking. J. Sol. Energy Eng..

[28] Yan J., Peng Y., Cheng Z. (2018). Optimization of a discrete dish concentrator for uniform flux distribution on the cavity receiver of solar concentrator system. Renew. Energy.

[29] Devarajan Y., Nagappan B., Subbiah G., Kariappan E. (2021). Experimental investigation on solar-powered ejector refrigeration system integrated with different concentrators. Environ. Sci. Pollut. Res..

[30] Malwad D., Tungikar V. (2020). Experimental performance analysis of an improved receiver for Scheffler solar concentrator. SN Appl. Sci..

[31] Shatar N.M., Sabri M.F.M., Salleh M.F.M., Ani M.H. (2023). Energy, exergy, economic, environmental analysis for solar still using partially coated condensing cover with thermoelectric cover cooling. J. Clean. Prod..

[32] Munir Z., Roman F., Niazi B.M.K., Mahmood N., Munir A., Hensel O. (2023). Thermal analysis of a solar latent heat storage system using Scheffler concentrator for agricultural applications. Appl. Therm. Eng..

[33] Farooq M. (2022). Thermal performance enhancement of nanofluids based parabolic trough solar collector (NPTSC) for sustainable environment. Alex. Eng. J..

[34] Yusof M.F. (2022). Clean water production enhancement through the integration of small-scale solar stills with solar dish concentrators (SDCs)—a review. Sustainability.

[35] El Ouederni A.R., Ben Salah M., Askri F., Ben Nasrallah M., Aloui F. (2009). Experimental study of a parabolic solar concentrator. J. Renew. Energies.

[36] Haddad Z., Abid C., Oztop H.F., Mataoui A. (2014). A review on how the researchers prepare their nanofluids. Int. J. Therm. Sci..

[37] Zhu D., Li X., Wang N., Wang X., Gao J., Li H. (2009). Dispersion behavior and thermal conductivity characteristics of Al2O3–H2O nanofluids. Curr. Appl. Phys..

[38] Kalaiselvam S., Parameshwaran R., Harikrishnan S. (2012). Analytical and experimental investigations of nanoparticles embedded phase change materials for cooling application in modern buildings. Renew. Energy.

[39] Khanjari Y., Kasaeian A.B., Pourfayaz F. (2017). Evaluating the environmental parameters affecting the performance of photovoltaic thermal system using nanofluid. Appl. Therm. Eng..

[40] Dumka P., Mishra D.R. (2020). Performance evaluation of single slope solar still augmented with the ultrasonic fogger. Energy.

